# Hydroxychloroquine partially prevents endothelial dysfunction induced by anti-beta-2-GPI antibodies in an in vivo mouse model of antiphospholipid syndrome

**DOI:** 10.1371/journal.pone.0206814

**Published:** 2018-11-06

**Authors:** Geoffrey Urbanski, Antoine Caillon, Caroline Poli, Gilles Kauffenstein, Marc-Antoine Begorre, Laurent Loufrani, Daniel Henrion, Cristina Belizna

**Affiliations:** 1 UMR CNRS 6015—INSERM U1083, MITOVASC Institute, Université d’Angers, Faculté de Médecine, Bâtiment IRIS 2, Angers, France; 2 Service de Médecine Interne et Maladies Vasculaires, Centre Hospitalier Universitaire, Angers, France; 3 Laboratoire d’immunologie et d’allergologie, Centre Hospitalier Universitaire, Angers, France; 4 Centre vasculaire et de la coagulation, Centre Hospitalier Universitaire, Angers, France; Weizmann Institute of Science, ISRAEL

## Abstract

**Background:**

Antiphospholipid syndrome is associated with endothelial dysfunction, which leads to thrombosis and early atheroma. Given that hydroxychloroquine has anti-thrombotic properties in lupus, we hypothesized that it could reduce endothelial dysfunction in an animal model of antiphospholipid syndrome. We evaluated the effect of hydroxychloroquine in preventing endothelial dysfunction in a mouse model of antiphospholipid syndrome.

**Methods:**

Antiphospholipid syndrome was induced by an injection of monoclonal anti-beta-2-GPI antibodies. Vascular reactivity was evaluated in mesenteric resistance arteries isolated from mice 3 weeks (APL3W) after receiving a single injection of anti-beta-2-GPI antibodies and after 3 weeks of daily oral hydroxychloroquine treatment (HCQ3W) compared to control mice (CT3W). We evaluated endothelial dysfunction by measuring acetylcholine-mediated vasodilation. A pharmacological approach was used to evaluate NO synthase uncoupling (tetrahydrobiopterin) and the generation of reactive oxygen species (Tempol).

**Results:**

Impaired acetylcholine-mediated dilation was evidenced in mice 3 weeks after anti-beta-2-GPI antibodies injection compared to CT3W, by reduced maximal dilation (p<0.0001) and sensitivity (pKd) (p = 0.01) to acetylcholine. Hydroxychloroquine improved acetylcholine-dependent dilation, on pKd (p = 0.02) but not maximal capacity compared to untreated mice. The addition of tetrahydrobiopterin (p = 0.02) and/or Tempol (p = 0.0008) improved acetylcholine-mediated dilation in APL3W but not in HCQ3W.

**Conclusions:**

We demonstrated that endothelial dysfunction in mouse resistance arteries persisted at 3 weeks after a single injection of monoclonal anti-beta-2-GPI antibodies, and that hydroxychloroquine improved endothelium-dependent dilation at 3 weeks, through improvement of NO synthase coupling and oxidative stress reduction.

## Introduction

The current classification criteria of antiphospholipid syndrome (APS) requires at least one clinical manifestation, thrombosis or pregnancy morbidity, in the presence of at least one serological assay, anticardiolipin antibodies (aCL), anti-beta-2-GPI antibodies (aB2GPI) and lupus anticoagulant, on 2 occasions 12 weeks apart [1].

Beta-2-GPI (B2GPI) seems to be the most relevant target of antiphospholipid antibodies (aPL) [2]. The epitopes of B2GPI are largely preserved along the evolution with a very high interspecies homology [3,4]. B2GPI interacts with multiple cells (including monocytes, platelets, trophoblasts and endothelial cells (EC)[5]) and coagulation factors [6]. The binding of antibodies against B2GPI has been shown to activate EC in mouse models of APS [7] and APS patients, inducing a proinflammatory and procoagulant phenotype in EC via up-regulation of adhesion molecules and cytokines, known as endothelial dysfunction (ED) [8]. In APS, ED promotes early atheroma [9], but also creates conducive ground for thrombosis [8]. ED is characterized by a reduction of the bioavailability of endothelium-derived vasodilators, mainly nitric oxide (NO), whereas contracting factors are increased, leading to an impairment of vasodilation [10]. Nitric oxide plays a major role in vascular homeostasis, regulating many processes such as leukocyte adhesion, thrombosis, EC migration and proliferation, vascular permeability, and vascular smooth muscle cell (SMC) growth and migration [11]. Ramesh et al. have shown that antiphospholipid antibodies (aPL) inhibit endothelial NO synthase (eNOS), with the reduced NO production causing leukocyte–EC interaction and thrombus formation. Consequently, eNOS antagonizing seems to be a critical initiating process in the pathogenesis of vascular manifestations of APS [12].

Many conditions must be met to preserve eNOS function. Among these, the bioavailability of tetrahydrobiopterin (BH4) seems crucial [13]. BH4 is an essential cofactor of NOS and a scavenger for reactive oxygen species (ROS) [13]. Consequently, BH4 is a key regulator of NO/ROS balance [14]. Cosentino et al. have demonstrated that oral BH4 supplementation reduces oxidative stress and restores endothelial function in patients with hypercholesterolemia [15].

Hydroxychloroquine (HCQ) is an essential drug in treating systemic lupus erythematosus (SLE) [16] and its use is also discussed in many other conditions including vasculitis [17]. HCQ has been shown to have anti-thrombotic effects in SLE patients with or without aPL [18,19] but has remained controversial in primary APS [20,21]. HCQ inhibits aB2GPI binding to phospholipid bilayers [22]. HCQ has also been shown to reduce thrombus size in an animal model of APS [23].

Most animal models of APS focus on experimental thrombosis through mechanical [24–27], laser-induced [28,29] or photochemical [30] vascular injury but few studies directly assess mechanical endothelial functions. We have previously evidenced impaired endothelium-dependent relaxation in mice after a one-week treatment with aB2GPI antibodies (from aPL-secreting hybridomas) [31]. This dysfunction was linked to decreased NO and the bioavailability of vasodilator prostanoids, and improved by the administration of aspirin or statin [31].

In this study, we evaluated endothelial-dependent vasorelaxation in mesenteric resistance arteries in C57Bl/6 mice treated with a single intraperitoneal injection of monoclonal aB2GPI to assess ED at 3 weeks, the influence of sex and the benefits of daily treatment with HCQ.

## Materials and methods

### Animal model of APS

The protocol was approved by the ethics committee for animal experimentation des Pays-de-la-Loire (No. CEEA.2011.13). Twelve-week-old C57Bl6 (male and female) mice were used (n = 10 per group, Janvier®, France).

### Immunization with aB2GPI and control antibodies

The mice were injected intraperitoneally with 150 μg of monoclonal recombinant anti-human B2GPI IgG1 mice antibodies (Sinobiological Laboratories, Beijing, China, ref. 11221-MM06) or with a monoclonal IgG1 control isotype (BioXCell laboratories, NH, USA, ref. BE0083). The endotoxin level was less than 0.1 EU/mg. Animals were sacrificed at 3 weeks after the administration of antibodies, by inhalation of CO2 (3 L/min). Blood samples were collected from the left ventricule and the mesentery were extracted.

### Measurement of aB2GPI, aCL and lupus anticoagulant activities

aB2GPI were quantified in the anti-B2GPI reagent and in the sera of mice by an enzyme-linked immunosorbent assay (ELISA). Wells (NuncMaxisorp, Nunc A/S, Thermo Fisher Scientific, Roskilde, Denmark) were coated overnight at 4°C with 100 μl of 10 μg/ml of recombinant murine B2GPI (R&D systems laboratories, MN, USA, ref. 6575-AH-050) or human B2GPI (Sinobiological Laboratories, Beijing, China, ref. 11221-H08H) in PBS. After saturation with washing buffer (WB) (0.05% Tween-20 in PBS, pH 7.4) containing 10% BSA (w/v), plates were successively incubated with monoclonal recombinant anti-human Fab B2GPI IgG1 mice antibodies (Sinobiological Laboratories, Beijing, China, ref. 11221-MM06) (increasing concentrations from 0.1 to 20 μg/ml), or monoclonal IgG1 control isotype (BioXCell laboratories, NH, USA, ref. BE0083) (increasing concentrations from 0.1 to 20 μg/ml) or mice serum (1:100) prepared in dilution buffer (DB) (0.1% BSA in WB, pH 7.4), and then with polyclonal rabbit anti-mouse IgG biotinylated immunoglobulins (Dako, CA, USA, Ref. E0413). Bound mAbs were revealed with the 3,3′,5,5′-tetramethylbenzidine substrate (Sigma-Aldrich, St Quentin Fallavier, France, Ref. T0440). Results are expressed in optical density (OD) 450 nm values after subtraction of the values obtained with polyclonal rabbit anti-mouse IgG biotinylated immunoglobulins alone.

Anti-cardiolipin activities (IgG, IgA and IgM) were determined using commercially available ELISA kit (Calbiotech, CA, USA, CA077T) as per the manufacturer’s instructions.

The LA was detected following the updated guidelines of the International Society on Thrombosis and Haemostasis, using a panel of two tests including activated cephalin time and dilute Russell’s viper venom time for screening and confirmation [32]. We tested the LA activity by comparing results with and without the addition of the monoclonal recombinant anti-human B2GPI IgG1 mice antibodies (20μg/ml) or the monoclonal IgG1 control isotype (20μg/ml) in healthy controls plasma.

### HCQ quantification

The HCQ quantification in mice serum was achieved using a liquid chromatography coupled with a UV detection (LC/UV) method. Chloroquine was used as the internal standard. Separation was performed on the EQUISORB C8 (150*4.6 mm, 5 μm) column after liquid/liquid extraction procedure. The mobile phase (phosphate buffer/acetonitrile, 85/15, v/v) is delivered at a flowrate of 1.5 ml/min. The HCQ and internal standard were detected at 340 nm. The method is fully approved according to the FDA recommendations. Results were expressed in mUA.

### Mice groups

Mice were divided into different experimental groups: mice sacrificed 3 weeks ("APL3W") after receiving aB2GPI, mice sacrificed 3 weeks after receiving control antibodies ("CT3W"), and mice sacrificed 3 weeks after receiving aB2GPI and treated with HCQ (20 mg/kg, daily gavage) ("HCQ3W"). If there were no differences between males and females, we planned to pool the results.

### Vascular reactivity of isolated mesenteric resistance arteries

The mesentery was removed and placed in an ice-cold physiological salt solution (PSS) (in mmol/L: 135.0, NaCl, 15.0, NaHCO3, 4.6 KCl, 1.5, CaCl2, 1.2, MgSO4, 11.0, glucose, 10.0, N-2-hydroxyethylpiperazine-N-2-ethylsulfonic acid). The PSS was maintained at pH 7.4, PO2 160 mmHg, PCO2 37 mmHg. Mesenteric resistance arteries were dissected and divided into 2-mm segments, subsequently mounted in a 4-chamber wire myograph (model 610M, DMT, Dk) as previously described [33]. An average of four arterial segments was calculated, providing one value per mouse.

First, vasoconstriction with potassium chloride (KCl) (80 mmol/L) was measured to determine vessel viability. After precontraction with phenylephrine to 70% of the maximal contractile response [34], cumulative concentration-response curves (CRC) in response to acetylcholine (ACh) (10^−9^ to 10^−5^ mol/L) and sodium nitroprusside (SNP) (10^−9^ to 10^−5^ mol/L) were performed. Alternatively, cumulative CRC to ACh were obtained before and after incubation (20 minutes) with BH4 and L-arginin, and with a combination of superoxide dismutase mimetic Tempol plus Catalase. To assess the effect of time and repeated ACh stimulations on the quality of artery relaxation, we performed “time control” experiments (n = 12), without drugs. Changes in relaxation due to time were used to correct the corresponding CRC to ACh.

### Statistical analysis

Results were expressed as mean±SEM. CRC were created using GraphPad Software (La Jolla, CA, USA). Sensitivity was expressed as pKd (= −log efficient concentration 50%) and potency as Emax. The Mann-Whitney U Test was used to compare groups and the Wilcoxon signed-rank test was applied to paired samples. P values below 0.05 were considered significant.

## Results

### Measurement of aB2GPI and aCL by ELISA, and the LA activity. Hydroxychloroquine quantification

**Fig 1:**
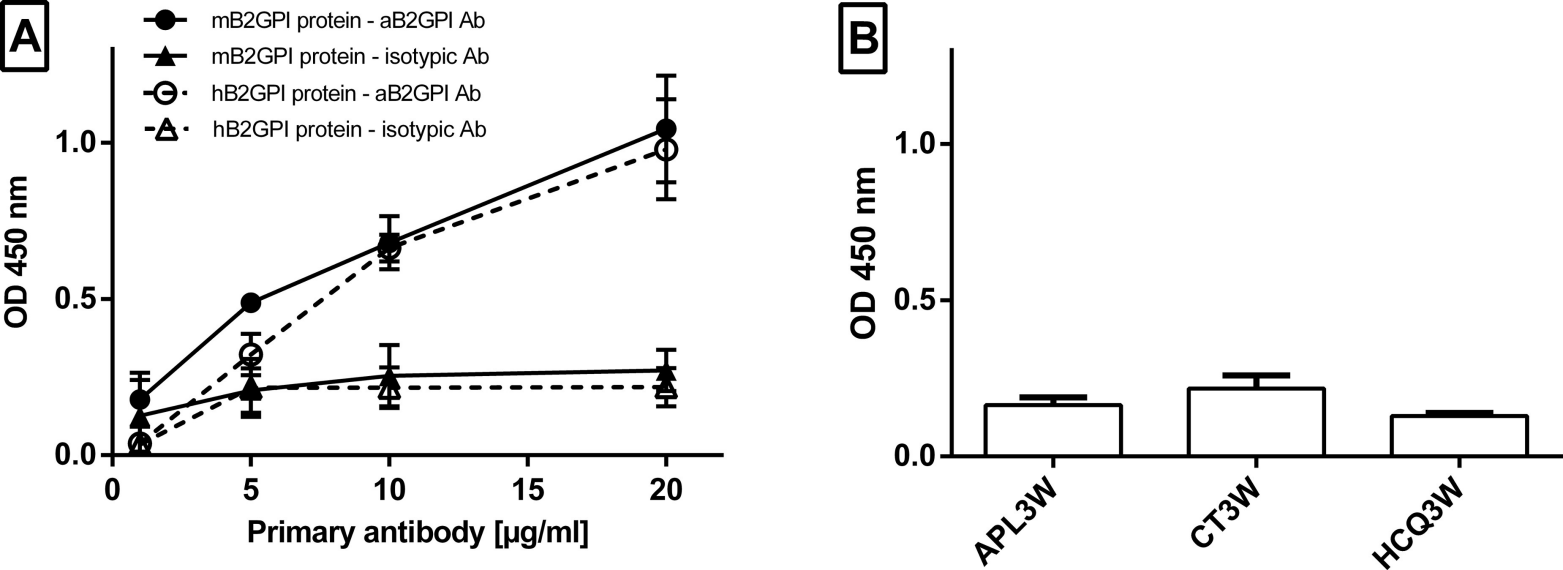
Anti-beta-2-GPI antibodies detection by enzyme-linked immunosorbent assay (ELISA). (A) Anti-human B2GPI mice antibodies and monoclonal IgG1 control isotype antibodies (from 1 to 20 μg/ml) were incubated with murine and human beta-2-GPI proteins (B) Anti-beta-2-GPI antibodies were assessed by ELISA in mice sera of APL3W, CT3W and HCQ3W groups (n = 3 for each, dilution 1:100). Footnotes: Values are expressed as mean±SEM. mB2GPI: murine beta-2-GPI protein, hB2GPI: human beta-2-GPI protein, aB2GPI Ab: monoclonal recombinant anti-human beta-2-GPI mice antibodies; isotypic Ab: monoclonal control isotype antibodies; OD: optical density.

Anti-human aB2GPI binding to murine B2GPI protein was similar to binding to human B2GPI protein and dose-dependent (Fig 1). We did not detect aB2GPI in mice sera in APL3W, CT3W and HCQ3W groups (n = 3), as OD for each groups were not higher than isotype controls (means±SEM: APL3W 0.16±0.03, CT3W 0.22±0.04 and HCQ3W 0.13±0.01).

**Fig 2:**
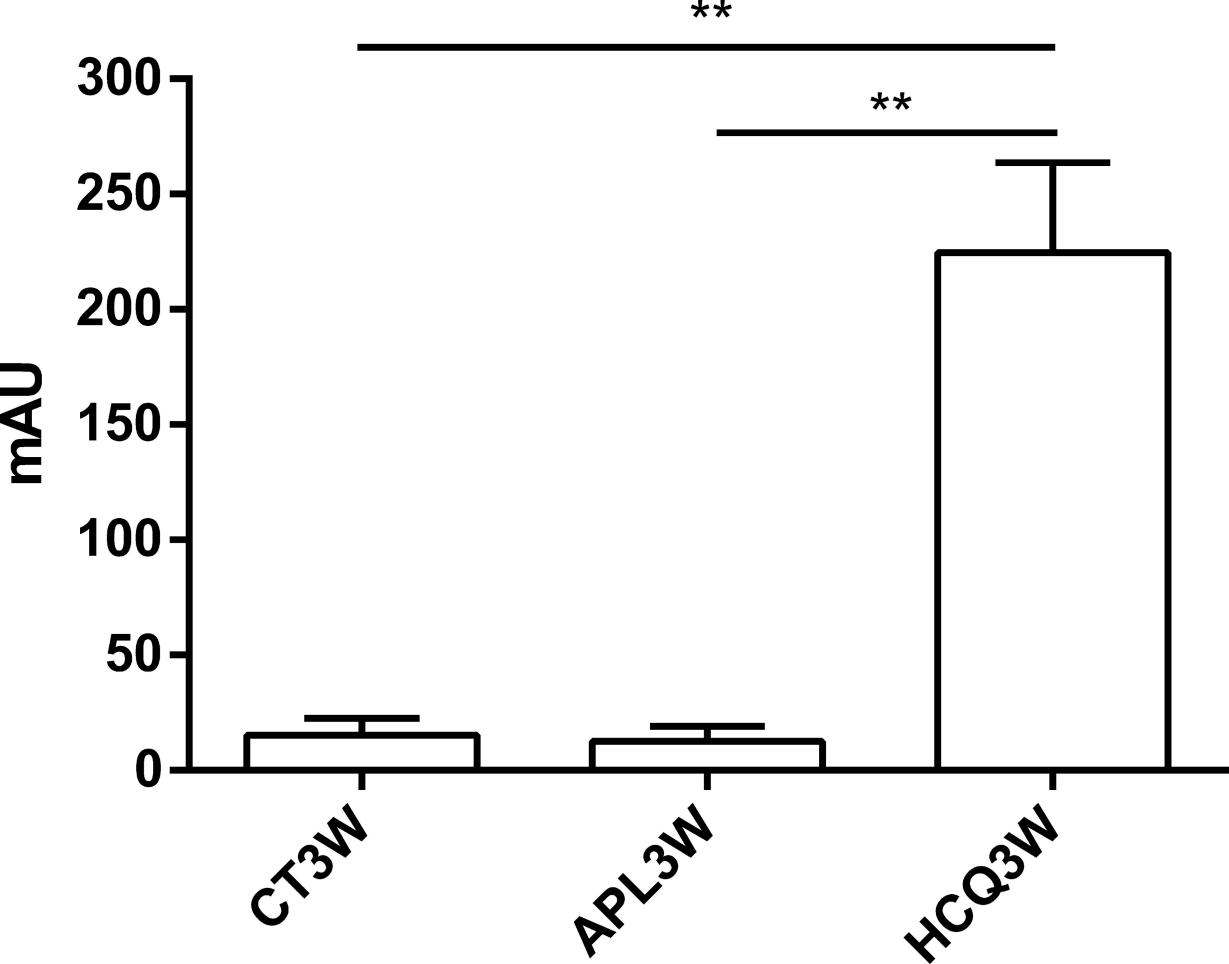
Hydroxychloroquine quantification in serum from APL3W, CT3W and HCQ3W mice. The serum HCQ quantification was achieved using a liquid chromatography coupled with UV detection method in mice sacrificed 3 weeks (APL3W, n = 3) after receiving aB2GPI, in mice sacrificed 3 weeks after receiving control antibodies (CT3W, n = 3), and in mice sacrificed 3 weeks after receiving aB2GPI and treated with HCQ (HCQ3W, n = 10). The serum concentration of HCQ3W mice were compared to APL3W (p = 0.007) and to CT3W (p = 0.007). Footnotes: The serum concentration is expressed as mean±SEM in mAU. Symbols show statistical significance in this order: single symbol (*) for 0.01<p<0.05, double symbol (**) for 0.001<p≤0.01 and triple symbol (***) for p≤0.001.

Anti-human aB2GPI (20μg/ml) did not cross react to cardiolipin, and anti-cardiolipin antibodies were not detected in mice sera (APL3W, CT3W and HCQ3W groups, n = 3). Neither the anti-human aB2GPI (20μg/ml) nor the IgG1 control isotype (20μg/ml) displayed LA activity. HCQ was significantly detected in mice serum in HCQ3W but neither in APL3W nor in CT3W (Fig 2).

### Endothelial function is impaired 3 weeks after injection of aB2GPI

**Fig 3:**
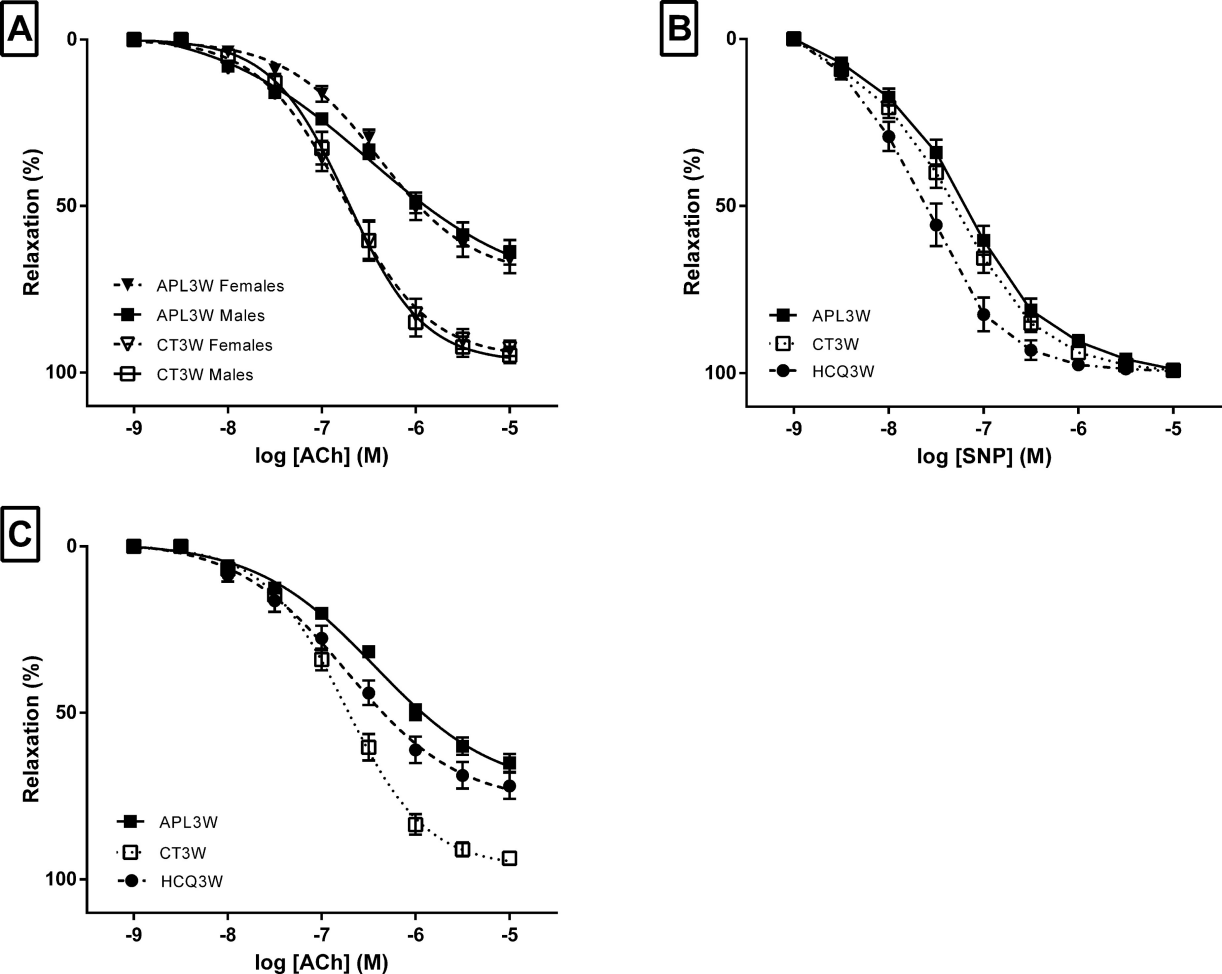
Acetylcholine- and sodium nitroprusside-dilation in APL3W, CT3W and HCQ3W. **Influence of sex.** Effects of monoclonal anti-beta-2-GPI antibody (aB2GPI) on dilation responses expressed as percentage of relaxation to acetylcholine (ACh) and sodium nitroprusside (SNP) in mice mesenteric arteries. Concentration-response curves were induced by increasing concentrations of ACh in mesenteric arteries isolated from mice sacrificed at 3 weeks after the peritoneal injection of monoclonal aB2GPI antibody or control antibody, with or without oral treatment with hydroxychloroquine (HCQ). (A) Results with ACh for females (n = 10) and males (n = 10) in CT3W and APL3W. (B) Results with SNP for mice infused with control antibodies (CT3W, n = 20) or aB2GPI (APL3W, n = 20), and for mice infused with aB2GPI and treated with HCQ mice (HCQ3W, n = 10). (C) Results with ACh for mice infused with control antibodies (CT3W, n = 20) or aB2GPI (APL3W, n = 20), and for mice infused with aB2GPI and treated with HCQ mice (HCQ3W, n = 10). Footnotes: Values are expressed as mean±SEM. Results related to females are shown by a broken line and to males by an unbroken line in panel A.

Arterial dilation of mesenteric resistance arteries in response to ACh was measured in the different experimental groups. Both potency (Emax 72.7%±3.1% vs 96.7%±1.2%; p<0.0001) and sensitivity (pKd 6.51±0.09 vs 6.71±0.06; p = 0.01) to ACh were reduced in APL3W compared to CT3W group (Table 1). Notably, no differences were observed in the ACh CRC between females and males (Fig 3A). Notably, CRC in response to SNP (endothelium independent dilation) did not differ between APL3W and CT3W. By contrast, sensitivity to SNP was better in HCQ3W compared to APL3W (pKd 7.65±0.12 vs 7.23±0.09 for HCQ3W and APL3W respectively; p = 0.019) but not to CT3W (pKd 7.65±0.12 vs 7.33±0.10 for HCQ3W and CT3W respectively; p = 0.07). (Fig 3B and Table 1).

**Table 1:** Potency (Emax) and sensitivity (pKd) of acetylcholine (ACh) and sodium nitroprusside (SNP) cumulative concentration response curves in APL3W, CT3W and HCQ3W.

	APL3W	CT3W	HCQ3W
n	20	20	10
ACh-mediated dilation			
Emax (%)	72.7 ± 3.1	96.7 ± 1.2 ***	76.3 ± 4.0
pKd	6.51 ± 0.09	6.71 ± 0.06 **	6.83 ± 0.13 †
SNP-mediated dilation			
Emax (%)	99.2 ± 0.3	99.6 ± 0.2	99.7 ± 0.2
pKd	7.23 ± 0.09	7.33 ± 0.10	7.65 0.12 †

Footnotes: All statistical comparisons are versus APL3W. Symbols show statistical significance in this order:

** for 0.001<p≤0.01 and triple symbol

*** for p≤0.001 for CT3W

† for 0.01<p<0.05 for HCQ3W. Values are expressed as mean±SEM.

### Endothelial function is partially restored in Hydroxychloroquine treated animals

ACh-mediated dilation was significantly improved by HCQ treatment (pKd = 6.83±0.13 vs 6.51±0.09 for HCQ3W and APL3W respectively; p = 0.02) (Fig 3C and Table 1). The restoration of dilation in the HCQ3W group reached an extent comparable to the CT3W group (pKd = 6.83±0.13 vs 6.71±0.06 for HCQ3W and CT3W respectively; p = 0.77). Maximal dilation was not modified (23.7%±4.0% vs 27.3%±3.1% for HCQ3W and APL3W respectively; p = 0.49). Notably, arteries from the HCQ3W group were more sensitive to SNP-dilation compared to the APL3W group (pKd = 7.65±0.12 vs 7.23±0.09 for HCQ3W and APL3W respectively; p = 0.02) but not to CT3W group (pKd 7.65±0.12 vs 7.33±0.10 for HCQ3W and CT3W respectively; p = 0.07) while maximal dilations were similar (Fig 3B and Table 1).

### Tetrahydrobiopterin and antioxidant in vitro mimic correction of endothelial dysfunction produced by Hydroxychloroquine treatment in vivo

**Fig 4:**
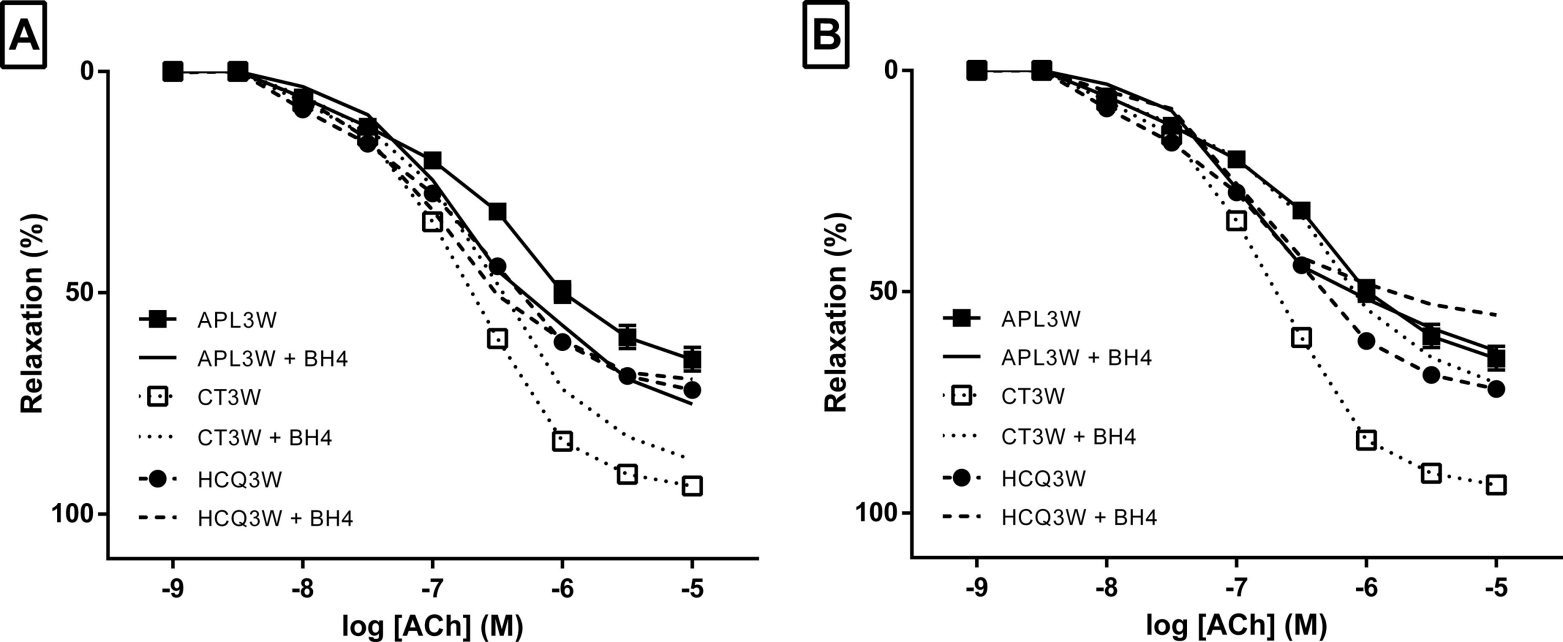
Effects of tetrahydrobiopterin and tempol on acetylcholine-dilation in APL3W, CT3W and HCQ3W. Effects of tetrahydrobiopterin (BH4) and Tempol on dilation responses expressed as percentage of relaxation to acetylcholine (ACh) in mice mesenteric arteries. ACh concentration-response curves were induced by increasing concentrations of ACh in mesenteric arteries, isolated from mice sacrificed at 3 weeks, infused with control antibodies (CT3W, n = 20) or anti-beta-2-GPI antibody (aB2GPI) (APL3W, n = 20), and in aB2GPI-treated mice with HCQ treatment (HCQ3W, n = 10). (A) Results with and without BH4. (B) Results with and without Tempol. Footnotes: Values are expressed as mean±SEM. Results without BH4/Tempol are presented with signs on lines. Results with BH4/Tempol are presented without sign on lines.

Adding BH4 improved ACh-dilation in the APL3W group (pKd = 6.65±0.06 vs 6.51±0.09 respectively with and without BH4 respectively; p = 0.02) (Table 2) while no changes were observed in the HCQ-treated. Maximal potencies of ACh-induced dilation were not affected by BH4 (Fig 4A).

**Table 2:** Potency (Emax) and sensitivity (pKd) of acetylcholine (ACh) cumulative concentration response curves without agent, with tetrahydrobiopterin (BH4)/L-arginin and with Tempol/Catalase in APL3W, CT3W and HCQ3W.

	APL3W	CT3W	HCQ3W
n	20	20	10
ACh-mediated dilation without agent			
Emax (%)	72.7 ± 3.1	96.7 ± 1.2	76.3 ± 4.0
pKd	6.51 ± 0.09	6.71 ± 0.06	6.83 ± 0.13
			
ACh-mediated dilation + BH4			
Emax (%)	76.9 ± 3.2	95.8 ± 2.2 †	70.9 ± 6.3
pKd	6.65 ± 0.06 *	6.46 ± 0.11	6.95 ± 0.16
			
ACh-mediated dilation + Tempol			
Emax (%)	64.1 ± 4.0	83.3 ± 5.3 ††	70.2 ± 7.8
pKd	6.88 ± 0.05 ***	6.32 ± 0.09 ††	6.96 0.08

Footnotes: Statistical comparisons of groups with BH4 or Tempol are versus the same group without agent. Symbols show statistical significance in this way

* for 0.01<p<0.05 and

*** for p≤0.001 for APLW

† for 0.01<p<0.05 and

†† for 0.001<p≤0.01 for CT3W. Values are expressed as mean±SEM.

Tempol also improved ACh-dilation in the APL3W group (pKd = 6.88±0.05 vs 6.51±0.09 with and without Tempol respectively; p = 0.0008) (Table 2). Tempol treatment did not modify relaxation in arteries in the HCQ3W (Fig 4B).

## Discussion

ED in APS represents a precocious feature of endothelial and platelet impairment, contributing to thrombosis [35] and early atheroma, associated with increased mortality and morbidity [36]. The ED induced by aPL antibodies has been largely investigated with biological markers [11,37–40]. In animal models, in vivo experiments often only focus on thrombogenesis [28,30] but few studies investigate vascular reactivity. We have evidenced a decreased vasodilation of resistance arteries in a mouse model of APS one week after injection of aB2GPI [31]. In this study, we evaluated ED 3 weeks after a single injection of monoclonal aB2GPI and tested the effects of daily oral administration of HCQ.

This study evidenced a persistent impairment of both maximal vasodilation and sensitivity to ACh 3 weeks after a single injection of monoclonal aB2GPI compared to control mice. Given the persistent impairment in endothelial function, our in vivo model may represent a valuable model of ED associated with APS, induced by monoclonal anti-B2GPI antibodies.

Although APS tends to affect mainly women [41], no differences in ACh-mediated dilation were found between male and female mice in our model. Notably, we used a single injection of aB2GPI, whereas in humans the circumstances leading to APS are part of a long immune process, probably influenced by sex.

Our major finding is that 3-week daily oral treatment with HCQ partly restored endothelium-dependent dilation in aPL-treated mice. Although HCQ has been shown to have an anti-thrombotic effect in SLE patients [13,14], its effects in primary APS remained controversial [20]. In experimental conditions, Virdis et al. demonstrated that HCQ could partially prevent ED in a mouse model of SLE and suggested that there was impact on oxidative stress [42].

In APS, decreased dilation could be caused by reduced NO bioavailability [43], which can result from NO scavenging by ROS or a lack of cofactor, a phenomenon known as “NOS uncoupling” [44]. Both BH4/L-Arginine and Tempol/catalase improved dilation in the APL3W group arteries, but had no effect in the HCQ-treated groups (HCQ3W). Importantly, the fact that neither BH4 nor Tempol could improve dilation in HCQ-treated animals implies that HCQ probably reduces ROS generation and therefore oxidative stress, as seen in lupus [45]. Oxidative stress seems to be a preliminary step of aPL pathogenicity, as demonstrated by Alves Delgado et al., with reduced paraoxonase activity and NO bioavailability [46]. NOS uncoupling also has a major role in atheroma and could be a potential target [47]. An oral and stable form of BH4 is available and could be an alternative in the event of APS. The hypothesis of ROS and BH4 interactions should not be forgotten, given that oxidation of BH4 (which produces BH2) by ROS is involved in vascular diseases [48]. However, this hypothesis does not explain the better response to SNP in HCQ-treated mice, which seems to be a specific effect of HCQ. Such a hypothesis requires further investigation.

In this mice model of APS, we did not detect aB2GPI in the blood of mice treated with the monoclonal recombinant anti-human B2GPI IgG1 mice antibodies 3 weeks after a single intraperitoneal injection. The lack of aB2GPI in blood stream below their usual half-life (IgG1) could mean that they are located on their targets (EC, platelets, monocytes) or that they have been degraded after the binding to their targets [37,49]. This could be a limitation to this study but it was not the aim of our work.

HCQ is used to treat SLE, however its use is still debated in the case of primary APS. In our mouse model, we started a 3-week oral HCQ treatment one day after aB2GPI injection, which could slow down the disease’s development, including antibodies binding inhibition on the phospholipid layer. This could be a limitation of our study given the fact that in clinics, HCQ is introduced when the disease is established.

In conclusion, this study validates our model of APS, which displays long-term endothelial dysfunction after a single injection of monoclonal aB2GPI. HCQ oral treatment improved endothelial function in APS mice, probably through the improvement of NOS coupling and the reduction of oxidative stress. These results create new possibilities for the treatment of primary APS.
